# Vascular and mesenchymal signatures of breast cancers associated with Yo and Ri paraneoplastic syndrome

**DOI:** 10.1186/s13058-026-02321-9

**Published:** 2026-06-17

**Authors:** Celeste Nicola, Elise Peter, Macarena Villagran-Garcia, Isabelle Treilleux, Géraldine Picard, Véronique Rogemond, Marine Villard, Jonathan Lopez, Bastien Joubert, Jérôme Honnorat, Valentin Wucher, Virginie Desestret

**Affiliations:** 1 Synaptopathies and Autoantibodies (SynatAc) Team, Institut NeuroMyoGène-MeLis, INSERM U1314/CNRS UMR 5284, Université de Lyon, Lyon, France; 2 French Reference Center On Paraneoplastic Neurological Syndromes, Hospices Civils de Lyon, Lyon, France; 3 Université de Lyon, Université Claude Bernard Lyon 1, Lyon, France; 4 Department of Biopathology, Centre Léon Bérard, Lyon, France

**Keywords:** Paraneoplastic neurological syndrome, Lymphangiogenesis, Lymph node metastasis

## Abstract

**Background:**

Paraneoplastic neurological syndromes (PNSs) are rare autoimmune diseases that occur in the context of cancer, but the mechanisms leading to immune tolerance breakdown remain unknown. Breast cancers (BCs) associated with Yo-PNS and Ri-PNS show distinct immune and genetic specificities, suggesting that tumour characteristics may contribute to autoimmunity. Herein, we aimed to investigate whether PNS-associated BCs display additional specificities, including stromal and vascular features that have not been systematically assessed.

**Methods:**

We compared 12 BCs associated with Yo-PNS (Yo-BCs) and 10 BCs associated with Ri-PNS (Ri-BCs) with two independent cohorts of non-PNS breast cancer controls (CtrlYo and CtrlRi) using tumour transcriptomic profiling with a targeted 760-gene panel and complementary immunohistochemistry.

**Results:**

Transcriptomic analysis identified 27 genes commonly differentially expressed in Yo-BCs and Ri-BCs compared with their respective controls, including upregulation of *FLT4*. Immunohistochemistry confirmed increased podoplanin-positive lymphatic structures in both Yo-BCs and Ri-BCs. In parallel, Yo-BCs showed predominant mesenchymal phenotype and downregulation of genes associated with hypoxia (*HIF1A*, *FLT1*, *LDHA*, *P4HA2*) compared with CtrlYo. These features were not present in Ri-BCs when compared with CtrlRi.

**Conclusion:**

These findings not only highlight subtype-specificities of Yo- and Ri-associated BCs, but also identify lymphangiogenesis as a shared tumour-intrinsic feature that may be part of a cancer-specific context required to initiate or sustain autoimmunity in PNS.

**Supplementary Information:**

The online version contains supplementary material available at 10.1186/s13058-026-02321-9.

## Background

Paraneoplastic neurological syndromes (PNSs) are rare autoimmune complications associated with cancer. They are characterised by an immune response directed against neuronal antigens ectopically expressed by the tumour. Among PNSs, Yo-PNS is frequently associated with breast cancer (BC) [1] and is characterised by anti-Yo autoantibodies, which target cerebellar degeneration-related protein 2 (CDR2) and its paralog CDR2L [2–4]. Anti-Ri autoantibodies, targeting the neuron-specific RNA-binding proteins NOVA1 and NOVA2, are the second most frequent paraneoplastic autoantibodies associated with BCs [5, 6]. Previous work revealed that BCs associated with Yo-PNS (Yo-BCs) are predominantly positive for the human epidermal growth factor receptor 2 (HER2 +) and hormone receptor negative (HR-) [7], a rare profile representing only 3.2% of BC cases overall [8]. In contrast, Ri-BCs are mainly HER2-/HR + luminal B BCs [9]. Moreover, molecular alterations (mutation or gain) in *CDR2* and *CDR2L*, potentially enhancing the immunogenicity of Yo antigens, have been exclusively observed in Yo-BC [7], while *NOVA1/2* alterations have not been found in Ri-BC [9]. These findings raised the possibility that specific tumour characteristics, beyond neural antigen alterations, could contribute to the immune tolerance breakdown in BC associated with PNS. Notably, a specific immune context, characterised by a shared B cell-dominated infiltration in Yo- and Ri-BC and an increased lymphatic dissemination have been reported in PNS patients compared to those without PNS [7, 9]. This dissemination process is strongly dependent on tumour neovascularisation and stromal remodelling [10, 11], features that remain unexplored in PNS. Such alterations in the tumour vasculature could not only create a permissive environment for tumour dissemination but also exert immunomodulatory effect that may contribute to the breakdown of the immune tolerance [12]. To further explore these differences, we conducted a transcriptomic analysis of Yo-BC and Ri-BC compared with control BC samples, focusing on pathways related to the tumour microenvironment, immune response, and signalling. Herein, by exploring the tumour transcriptome in PNS-BC, we highlighted mesenchymal dynamics that, beyond antigenic alterations and specific immune infiltration pattern, may create a permissive context for immune tolerance breakdown.

## Methods

### Study design and tumour selection

**Fig. 1:**
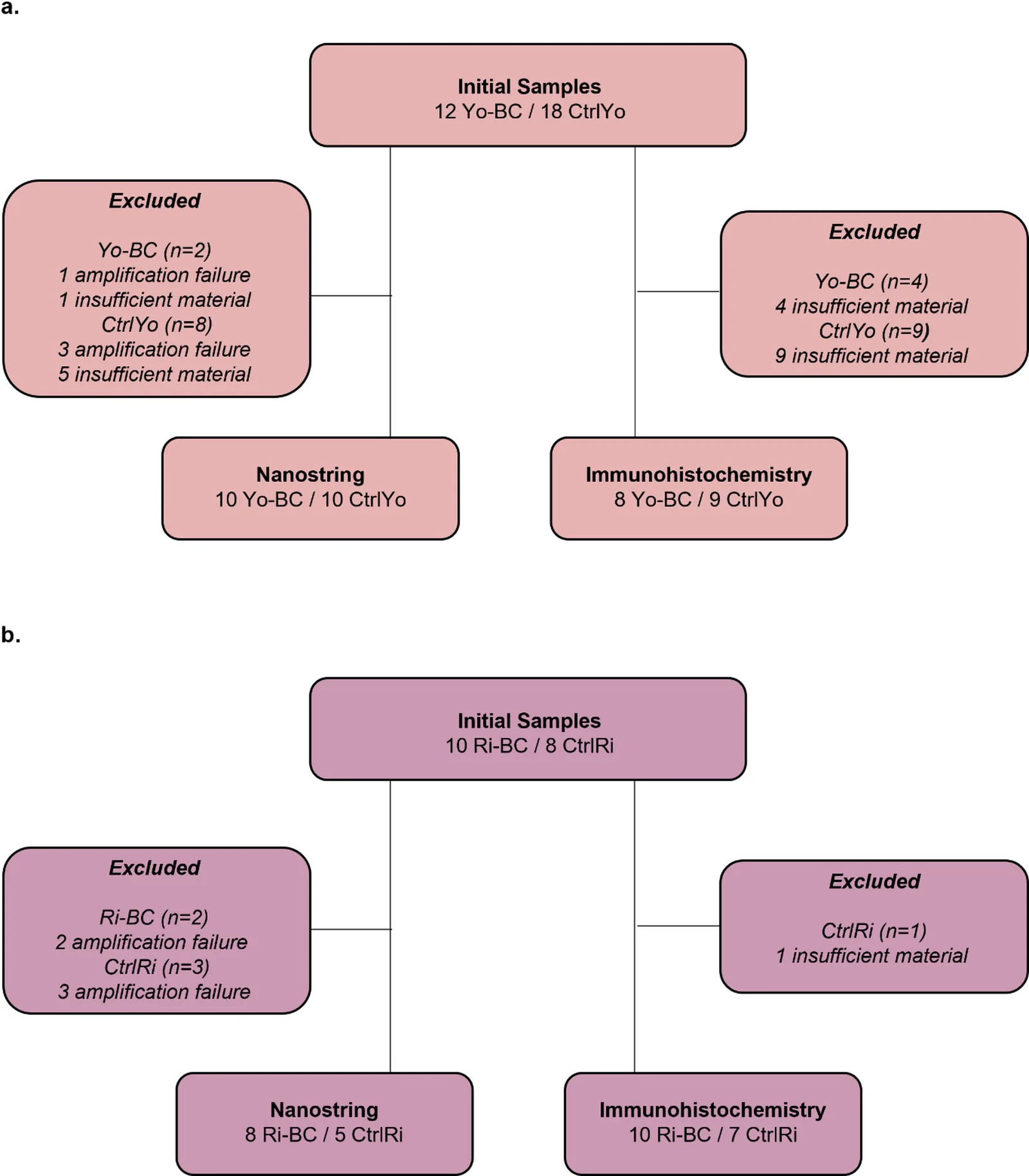
Flowchart of sample selection for transcriptomic (NanoString) and immunohistochemical analyses in the Yo-BC **a** and Ri-BC **b** cohorts and their respective controls BC (CtrlYo, CtrlRi). BC, breast cancer

Patients with Yo-PNS and with Ri-PNS associated with BC were identified retrospectively by the French Reference Center for PNS and autoimmune encephalitis (Lyon, France). Inclusion criteria as well as complete clinical description of these cohorts have been published elsewhere [7, 9]. Based on the availability of tumour samples deemed suitable for transcriptomic and immunohistochemical analyses, 12 primary Yo-BCs and 10 primary Ri-BCs were included. Control tumours (CtrlYo, n = 18; CtrlRi, n = 8) were subsequently selected from the biopathology department of the Centre Léon Bérard (BB-0033–00050, CRB Centre Léon Bérard, Lyon, France) based on HER2 status, HR status and lymph node status (N + /N0). Controls were selected to achieve comparable group-level distributions across these criteria. Due to amplification failure and insufficient tumour material, NanoString analysis (NanoString Technologies) was performed on 10 Yo-BCs, 10 CtrlYo, 8 Ri-BCs, and 5 CtrlRi. Immunohistochemistry (IHC) was performed on 8 Yo-BCs, 9 CtrlYo, 10 Ri-BCs, and 7 CtrlRi (Fig. 1). Tumour samples were collected at diagnosis or during the initial phase of disease management, prior to the initiation of systemic anticancer therapy in all but one patient (Ri-PNS group). Clinicopathologic characteristics of patients included in the present study are provided in Additional file 1: Table S1, and for the NanoString and IHC subsets separately in Additional file 2: Table S2.

### NanoString analysis

Gene-expression profiling was performed using a commercial 760 gene Tumor Signaling 360 Panel (NanoString Technologies). Total RNA was extracted from macrodissected FFPE tumour sections using the FormaPure RNA Extraction Kit (Beckman Coulter, Brea, CA, USA, Cat # C19157). The RNase-Free DNase Set (Qiagen, Hilden, Germany, Cat # 79,254) was used to remove DNA. The quantity and purity of the RNA was assessed with the NanoDrop Lite Spectrophotometer (Thermo Fisher Scientific, Waltham, MA, USA, RRID: SCR_025369). In case of low 260/230 ratios (< 1.8) the samples were re-purified by chloroform and subsequent ethanol precipitation. The quality of the RNA was controlled using Agilent 2100 Bioanalyzer Instrument (Agilent Technologies, Santa Clara, CA, USA, RRID: SCR_018043) with either the RNA 6000 Pico or Nano Kit (Agilent Technologies, Cat # 5067–1514, Cat # 5067–1512). According to the manufacturer’s instructions, 100 ng total RNA was used; after hybridisation of the RNA following the Gene Expression CodeSet RNA Hybridization Protocol (NanoString Technologies), samples were washed and loaded on to the nCounter Cartridge (NanoString Technologies) and subsequently analysed using the nCounter Digital Analyzer (NanoString Technologies). NanoString nCounter quality control was assessed using nSolver software, including imaging quality (field of view > 75%), binding density (0.1–2.25), positive control linearity (R^2^ > 0.95), and limit of detection based on positive controls (0.5 fM above negative controls + 2 SD). All samples passed quality control criteria. Samples were distributed across cartridges in a balanced manner to minimise potential batch effects. Raw mRNA data were normalised using a set of 20 predetermined housekeeping genes within the nSolver Analysis Software (NanoString Technologies, RRID: SCR_003420). Normalised data are available in Additional file 3: Table S3. The Tumor Signaling 360 Panel (NanoString Technologies) includes pre-annotated genes grouped into three major biological domains (microenvironment organisation, immune response, and tumour signalling), each further divided into functionally defined categories, themselves divided into subcategories (see NanoString website for details). For forest plot representation, to allow functional grouping, genes with multiple annotations were manually re-assigned to the most representative domain solely for representation purpose (Additional file 4: Table S4).

### Immunohistochemistry

Expression of carbonic anhydrase IX (CA IX) and podoplanin (PDPN) was assessed by immunohistochemistry using specific antibodies (CA IX: Roche Diagnostics, Penzberg, Germany, Cat # 08313466001, RRID: AB_3716892; PDPN: Agilent Technologies France, Les Ulis, France Cat # M361929-2, RRID: AB_2162081) and an automated IHC protocol. Immunohistochemical staining was measured using a semi-quantitative approach and independently evaluated by two observers. In cases of discrepancy, slides were jointly reviewed to reach a consensus score.

For CA IX staining, a semi-quantitative additive score (CA IX score) was used, combining both staining intensity on tumour cells, scored from 0 (no staining) to 3 (high intensity) and myofibroblast staining extent, scored from 0 (absence of staining) to 3 (diffuse staining). The total score thus ranged from 0 to 6. Tumours were then categorised as either “low” and “high” using a cut-off score of ≥ 2. For PDPN, a value (PDPN score) was assigned according to the number of capillaries positive for staining; 0 for no capillaries and 3 for a high number of capillaries. Tumours were then categorised as negative (PDPN-) and positive (PDPN +) based on PDPN expression in intratumoural capillaries using a cut-off of ≥ 1.

### Statistical analysis

Gene expression levels were compared between Yo-BC and CtrlYo samples, and between Ri-BC and CtrlRi samples, using the Wilcoxon-Mann–Whitney test on NanoString gene expression data. Detailed gene-level results, including mean expression values, *log*_*2*_ Fold Changes, raw *p* values, and Benjamini–Hochberg adjusted *p* values (FDR), are provided in Additional file 5: Table S5. Pathway enrichment analysis was performed using the Panther 2016 database, which include 112 functional terms, using Enrichr (http://amp.pharm.mssm.edu/Enrichr) and applying Fisher’s exact test for overrepresentation with a threshold on raw *p* value < 0·05 for considering significant pathways. Immunohistochemical analysis was performed using a semi-quantitative scoring method and Fisher’s exact test to compare groups. Correlations between PDPN score and FLT4 transcript levels, was assessed using Spearman’s correlation test. The number of differentially expressed (DE) genes annotated in specific categories and subcategories was calculated for each group, and comparisons were performed using Fisher’s exact test. A *p* value < 0·05 was considered significant for all tests. P values are unadjusted. Statistical analyses were performed using R software (version 4.0.3; R Core Team, 2025, R Foundation for Statistical Computing, Vienna, Austria; https://www.R-project.org) and GraphPad Prism 6 software (GraphPad Software, San Diego, CA, USA).

## Results

### Shared tumour-related and microenvironment features involving angiogenesis

**Fig. 2:**
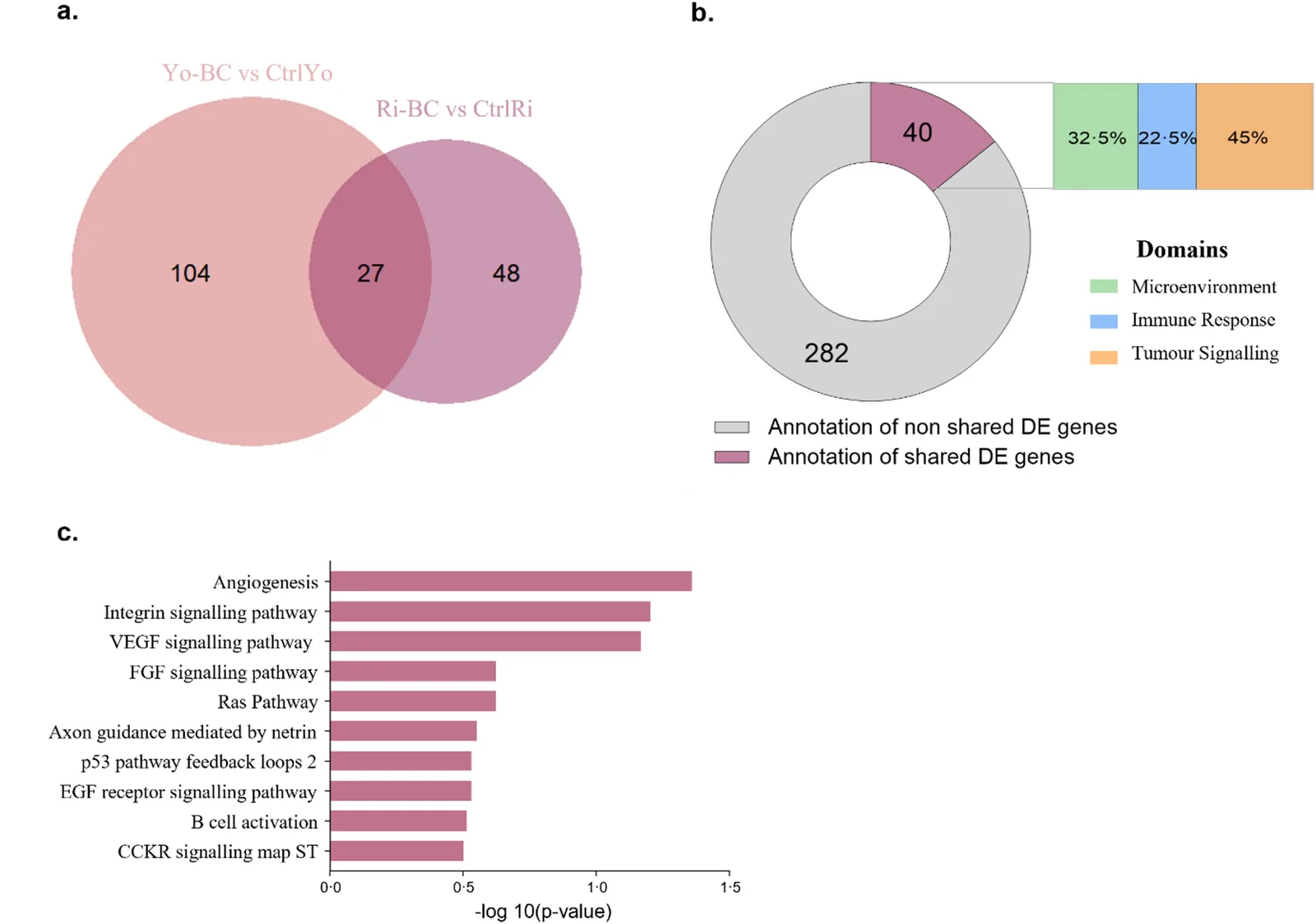
Shared transcriptomic signatures in Yo and Ri breast cancers. **a** Venn diagram showing the overlap between DE genes identified in Yo-BCs vs CtrlYo and Ri-BCs vs CtrlRi comparisons (areas not drawn to scale). **b** The donut chart shows the number of annotations corresponding to non-shared DE genes (grey, n = 282) and shared DE genes (purple, n = 40). The histogram displays the distribution of the 40 annotations associated with shared DE genes across the three NanoString domains: Tumour Signalling (45%, 18/40), Microenvironment (32.5%, 13/40), and Immune Response (22.5%, 9/40). **c** The top 10 pathways enriched by the 27 shared DE genes determined by the Panther database are presented in descending order based on the -log10 transformation of their *p* value (*p < 0·05; Fisher’s exact test). BC, breast cancer; DE, differentially expressed; CCKR, cholecystokinin receptor; EGF, epidermal growth factor; FGF, fibroblast growth factor; p53, tumour protein p53; ST, signal transduction; VEGF, vascular endothelial growth factor

Transcriptome profiling identified a total of 179 genes significantly DE between PNS-BC and their controls using nominal* p* values. Using Benjamini*–*Hochberg adjusted *p* values, three genes in Yo-PNS and none in Ri-PNS are significant (Additional file 5: Table S5). Comparing Yo-BC to CtrlYo we found 131 DE genes (88 upregulated, 43 downregulated, Additional file 6: Table S6), and comparing Ri-BC to CtrlRi we found 75 DE genes (57 upregulated, 18 downregulated, Additional file 7: Table S7); notably, 27 DE genes were shared between the two comparisons (Fig. 2a). Each DE gene were assigned to at least one annotation, resulting in a total of 322 annotations, including 40 annotations corresponding to the 27 shared DE genes (Additional file 4: Table S4). Among these 40 annotations of shared DE genes, 45% were related to tumour signalling, 32.5% to the microenvironment and 22.5% to immune response domain (Fig. 2b). To gain further insight into the involved molecular pathways, we performed pathway enrichment analysis (Fig. 2c). The top three shared enriched pathways were angiogenesis (*p* = 0.04), integrin signalling, and VEGF signalling (both *p* = 0.06), suggesting involvement of vascular and adhesion-related pathways in PNS-BC pathophysiology. These results highlight that, beyond immune specificities, PNS-associated BCs share tumour-related and microenvironment features, involving vascular processes.

### A common enhanced lymphangiogenesis

**Fig. 3:**
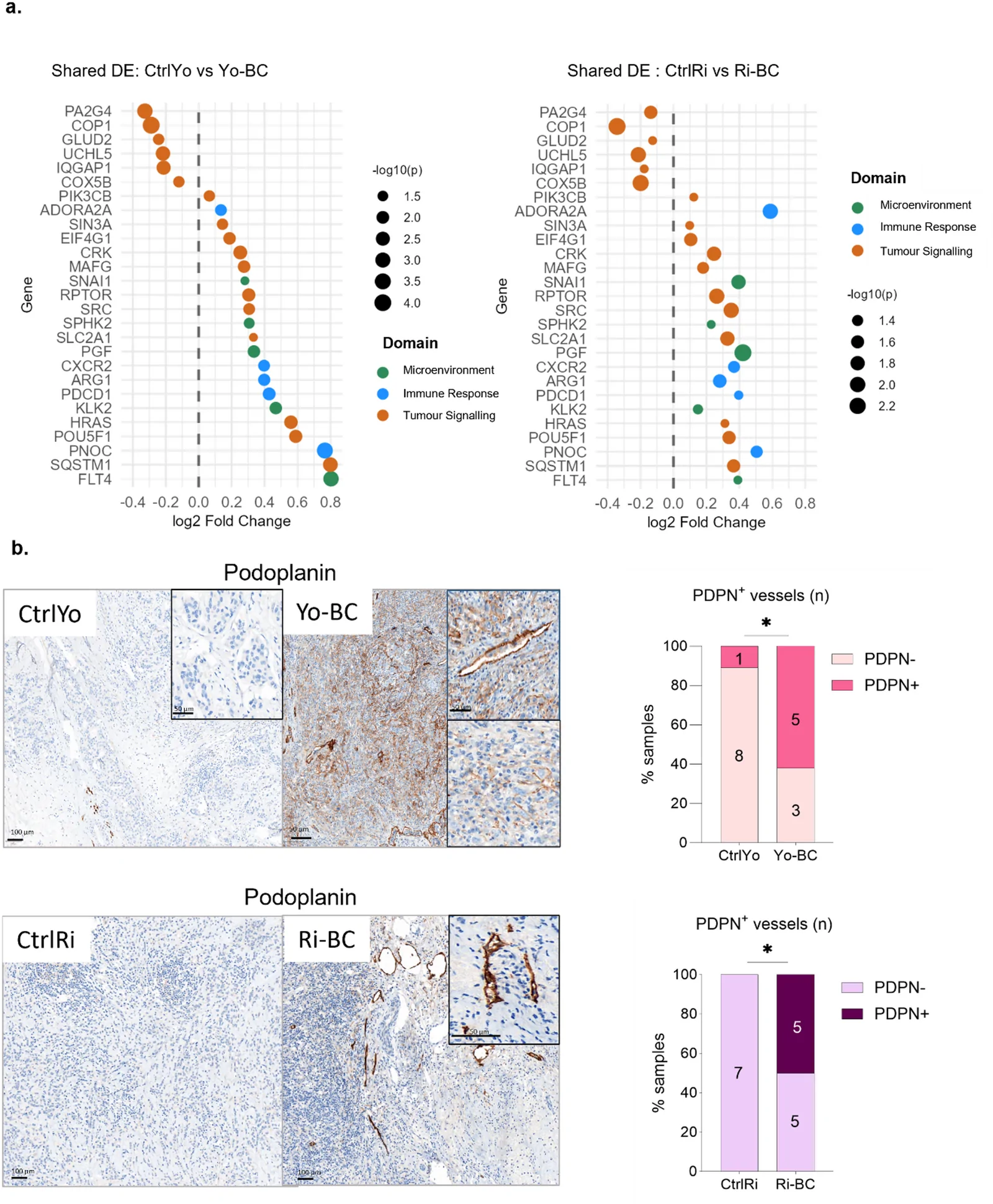
Expression of shared differentially expressed genes in Yo-BC and Ri-BC with a focus on lymphangiogenesis. **a** Dot plots showing the log2 fold changes of the 27 shared DE genes identified in both comparisons: Yo-BC vs CtrlYo (left) and Ri-BC vs CtrlRi (right). Dot size reflects statistical significance (*-log*_10_ *p* value; Wilcoxon-Mann–Whitney test). Gene colour indicates the NanoString domain: Tumour Signalling (orange), Immune Response (blue), and Microenvironment (green). Genes are organised in ascending order based on the absolute value of their log2fold change in the comparison Yo-BC vs CtrlYo. **b** Immunohistochemical measurement of PDPN in Yo-BCs, Ri-BCs compared to respective controls. Representative images show PDPN staining in Ctrl-Yo, Yo-BC, Ctrl-Ri, and Ri-BC. Insets illustrate high-magnification views where PDPN expression is observed in capillaries and stromal myofibroblasts (Yo-BC), or restricted to capillaries (Ri-BC). PDPN expression was assessed using a semi-quantitative scoring method (score 0–3) based on the number of PDPN-positive capillaries. Tumours were classified as PDPN + (score ≥ 1) or PDPN- (score = 0/0·5). Histograms summarise the proportion of PDPN + and PDPN- tumours, and the number of samples in each group is indicated (*p < 0·05; Fisher’s exact test). Scale bars: main images, 100 µm; insets, 50 µm. BC, breast cancer; DE, differentially expressed; PDPN, podoplanin

We then analysed the expression of the 27 shared DE genes between the two comparisons (Fig. 3a, Additional file 8: Figure S1). Among the microenvironment-related genes, there was upregulation of several vascular-associated genes in PNS-BC, including *FLT4* (encoding the lymphangiogenic receptor VEGFR3), *PGF* (encoding placental growth factor, a VEGF family member) and *SPHK2* (encoding sphingosine kinase 2, an enzyme involved in endothelial activation). Notably, *FLT4* was the most upregulated gene in Yo-BCs (log_2_Fc = 0·80; *p* = 0·0007) and remained highly upregulated in Ri-BCs (log_2_Fc = 0·39; *p* = 0·0481, Fig. 3a). We therefore analysed the expression of PDPN, a well-established marker of lymphatic endothelial cells by immunohistochemistry to assess the lymphatic vasculature in BC tissue samples. A semi-quantitative analysis found a significantly higher proportion of PDPN + tumours in both Yo-BC (5/8) and Ri-BC (5/10) groups compared to their respective controls (1/9 for CtrlYo and 0/7 for CtrlRi), indicating increased lymphatic density in BC-PNS (Fig. 3b). In addition, the PDPN score was positively correlated with *FLT4* transcript levels, suggesting that the PDPN score reflects the underlying lymphatic gene expression (Additional file 9: Figure S2).

Together, these findings indicate that BC-PNS tumours share a common enhanced lymphangiogenesis, which emerges as the shared vascular signature in Yo- and Ri-BC compared to controls.

### Shared tumour microenvironment remodelling, immune modulation, and metabolic reprogramming

Beyond lymphangiogenesis features, transcriptomic analysis revealed significant remodelling of the tumour stroma, evidenced by the upregulation of *KLK2* and *SNAI1*, key transcription factors involved in epithelial-to-mesenchymal transition (EMT) (Fig. 3a). The immune landscape showed a distinct immunoregulatory profile compared to control BC, with altered expression of immune checkpoint molecules (*PDCD1*, *ADORA2A*), immunomodulators (*PA2G4*, *EIF4G1*), myeloid-derived suppressor markers (*CXCR2*, *ARG1*), and a B-cell activity marker (*PNOC*). Tumour signalling pathways were characterised by the upregulation of genes promoting cell proliferation, including *POU5F1*, *HRAS*, *CRK* and *SRC*, alongside the downregulation of tumour suppressor genes such as *IQGAP1* (a modulator of TGF-β signalling) and *COP1* (a regulator of p53). Concomitantly, activation of the phosphoinositide 3-kinase (PI3K)/ mechanistic target of rapamycin (mTOR) pathway was indicated by increased expression of *PIK3CB*, *RPTOR*, and *SQSTM1*, suggesting enhanced growth and autophagy-related processes, whereas *UCHL5*, a deubiquitinase involved in mTOR regulation, was downregulated. At the metabolic level, transcriptional changes pointed toward a shift to glycolytic metabolism, with upregulation of *SLC2A1*, supporting increased glucose uptake, and downregulation of *GLUD2* and *COX5B*, both implicated in mitochondrial oxidative metabolism (Fig. 3a). These findings highlight a shared complex molecular reprogramming in PNS-BC, involving stromal remodelling, immune specificities, and metabolic shifts.

### Particular microenvironmental features and altered hypoxic signalling in Yo-BCs

**Fig. 4:**
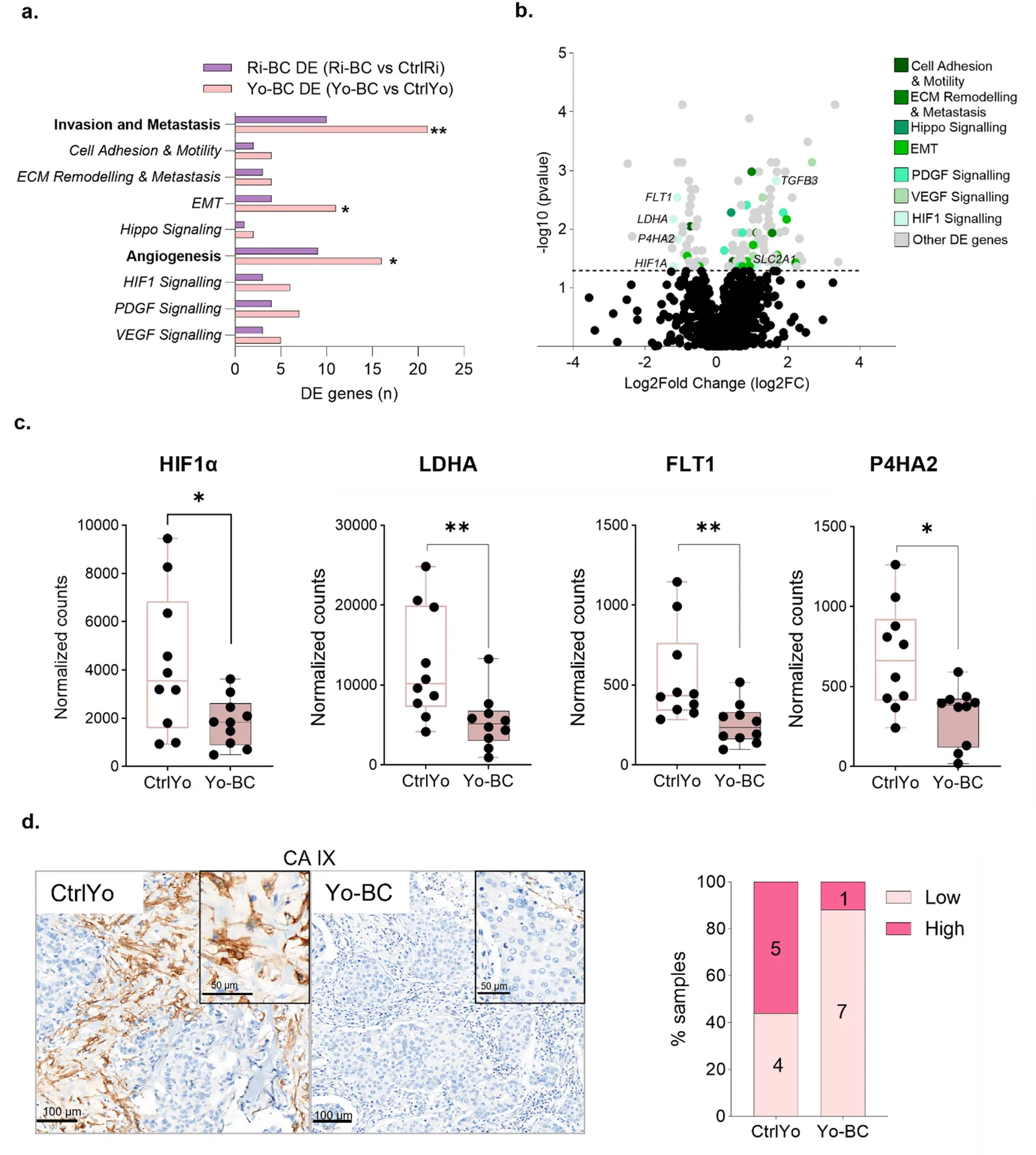
Mesenchymal signatures in Yo-BCs. **a** Histogram presenting the number of DE genes annotated to microenvironment domain in Yo-BC vs CtrlYo and Ri-BC vs CtrlRi comparisons. Genes were grouped in categories (in bold) and subcategories (in italics). Asterisks indicate categories or subcategories showing significant differences in the number of DE genes between the two comparisons (*p < 0·05; **p < 0·01; Fisher’s exact test). **b** Volcano plot showing gene expression changes (log2 fold change vs -log10 p value) for genes belonging to the microenvironment domain in Yo-BCs vs CtrlYo. Genes are coloured and according to their NanoString annotation. Genes included in the HIF1 Signalling subcategory are labelled. **c** Normalised mRNa expression counts for genes belonging to the HIF1 Signalling subcategory (*HIF1a*, *LDHA*, *FLT1*, and *P4HA2*), showing downregulation in Yo-BCs compared to CtrlYo. Box-and-whisker plots show median, minimum to maximum, and individual sample values (*p < 0·05; **p < 0·01; Wilcoxon-Mann–Whitney test). **d** Representative images showing immunohistochemical detection of CA IX in Yo-BC (n = 8) and CtrlYo (n = 9). Insets illustrate high-magnification views where CA IX expression is observed in myofibroblasts of CtlrYo and not in Yo-BC. CA IX was quantified using a semi-quantitative additive score, combining staining intensity on tumour cells and myofibroblast staining extent. Tumours were classified as CA IX-high (CA IX score ≥ 2) or CA IX-low, as summarised in histograms. Scale bars: main images, 100 µm; insets, 50 µm. CA IX, carbonic anhydrase 9; EMT, epithelial-mesenchymal transition; FLT1, Fms-related tyrosine kinase 1 (VEGFR1); HIF1α = hypoxia-inducible factor 1 alpha; LDHA, lactate dehydrogenase A; PA2HA2 = prolyl 4-hydroxylase subunit alpha 2; PDGF, platelet-derived growth factor; PNS, paraneoplastic neurological syndrome

Despite these shared features, a difference in the distribution of DE genes between Yo-BCs vs CtrlYo compared to Ri-BCs vs CtrlRi in the different domains was found (Fig. 4a, Additional file 10: Figure S3). In the Yo-BCs vs CtrlYo comparison, there were more DE genes involved in tumour inflammatory signalling pathways (e.g. *NF-κB*, *JAK-STAT*), oxidative stress responses (Nrf2 & Oxidative Stress), sustained proliferative signals (oestrogen, *PI3K-AKT*), genomic instability (Additional file 10: Figure S3a), and diverse immune cell-type signatures, including neutrophils, dendritic cells, and cytotoxic T cells, B cells, and exhausted CD8 + T cells (Additional file 10: Figure S3b). These were accompanied, notably, by a significantly higher number of DE genes related to the microenvironment domain (Fig. 4a). In particular, DE genes in the Activating Invasion and Metastasis category (especially the genes in the subcategory EMT) and Angiogenesis category were significantly more numerous in Yo-BCs vs CtrlYo compared to Ri-BCs vs CtrlRi. This suggests that Yo-BCs are characterised by increased expression of mesenchymal and EMT-related genes beyond the shared lymphangiogenic vascular phenotype described above in Yo- and Ri-BCs. EMT-related genes (except for *TTK* et *KDM1A*) were all upregulated in Yo-BC compared to CtrlYo (Fig. 4b). Concerning the Angiogenesis category in Yo-BCs, all the genes annotated in the PDGF Signalling and VEGF Signalling subcategories were upregulated in Yo-BCs compared to CtrlYo, while genes in the HIF1 Signalling subcategory were predominantly downregulated in Yo-BCs (4 of 6 genes). These included the master regulator of hypoxia, *HIF1α* and some of its target genes (*FLT1*, *LDHA*, and *P4HA2*; Fig. 4b and c). This was consistent with IHC semiquantitative assessment of the HIF1α-target CA IX, revealing a trend towards a lower frequency, even if non-significant (*p* = 0.13), of CA IX-high tumours in Yo-BCs (1/8) compared to CtrlYo (5/9), suggesting an atypical hypoxia status in Yo-BC (Fig. 4d). In Ri-BC, among the genes in the HIF1 signalling subcategory there were two DE genes (NOS3 was up and RBX1 was down; Additional file 11: Figure S4a), IHC semiquantitative assessment of CA IX revealed no significant difference in the frequency (*p* = 0.48) of CA IX-high tumours in Ri-BCs (8/10) compared to CtrlRi (7/7) (Additional file 11: Figure S4).

Taken together, these findings suggest that particular EMT-related transcriptional activity and altered hypoxic signalling may represent microenvironmental features characterising Yo-BCs.

## Discussion

In the present study we identified shared transcriptional features in BCs associated with Yo- and Ri-PNS, despite different histomolecular backgrounds. These observations suggest that Yo- and Ri-BCs may share a lymphangiogenic signature, accompanied by stromal remodelling and oncogenic metabolic shifts. Yo-BCs additionally displayed a mesenchymal transcriptional profile characterised by EMT-related gene expression and altered hypoxia-related signalling. This contributes to the ongoing effort to define intrinsic features of PNS-associated tumours by highlighting non-immune components that may be involved in tolerance breakdown.

*FLT4*, a gene coding for VEGFR-3, a key receptor on lymphatic endothelial cells activated by VEGF-C/D, was upregulated in Yo- and Ri-BCs when compared to their controls. Together with increased PDPN staining, in both vessels and stroma, this is consistent with enhanced lymphangiogenesis in PNS-associated tumours [13, 14]. Lymphatic remodelling therefore appears to be a recurrent feature in PNS-tumours and may be associated with anti-tumour immunity. Indeed, increased lymphangiogenesis has been proposed to promote antigen trafficking and immune cell infiltration, which may facilitate tumour-specific immune responses [12, 15, 16]. This is consistent with the marked B cell-dominated immune infiltration, previously reported in PNS-associated tumours [7, 9, 17, 18], as also reflected by the transcriptomic data reported herein. Such intense immune activation is often illustrated by the phenomenon of “burn-out” tumours [7, 9, 19] or the regression of imaging findings suggestive of malignancy [20]. Lymphangiogenesis has also been associated with the formation of intratumoural tertiary lymphoid structures (TLSs) which can support local adaptive immune responses [21–23]. In the context of PNS, this intratumoural priming may initiate or amplify humoral immunity against onconeural antigens. In line with this, TLSs harbouring CDR2L deposits have been described in Yo-associated tumours [7, 18] and, in ovarian teratomas from patients with anti-NMDA receptor encephalitis, TLSs harbour antigen-specific B cells undergoing affinity maturation have been reported [17]. Thus, lymphangiogenesis may contribute to the breakdown of immune tolerance in PNS-BCs by enabling the trafficking of immune cells, and by fostering the formation of structured niches that support humoral autoimmunity. However, TLSs were not directly assessed in the present study, and their contribution in this context therefore remains speculative. Taken together, these observations raise the possibility that lymphangiogenesis may contribute to immune tolerance breakdown in PNS-BCs, although direct mechanistic evidence is lacking.

Lymphatic vessels also provide routes for tumour cells dissemination [11, 24, 25]. This is in line with the frequent observation of lymph node involvement in PNS patients [19, 20, 26], including those with Yo- and Ri-BCs [7, 9]. Transcriptomic data reported herein suggest that BC cells in Yo- and Ri-associated tumours may exhibit features consistent with a more invasive phenotype. These include upregulation of EMT-related genes (*e.g. SNAI1*), activation of oncogenic signalling (*HRAS*, *SRC*), and metabolic shift toward aerobic glycolysis (*SLC2A1*), features that have been associated with increased proliferative state and metastatic potential [27–34]. However, these features are inferred from gene expression data and were not directly assessed functionally in this study. The combination of expanded lymphatic networks and transcriptional features associated with invasiveness may therefore be consistent with the higher burden of lymph-node involvement, although this relationship cannot be established causally from the present data. Additionally, disseminating tumour cells may directly carry tumour-associated antigens to the lymph nodes, thereby contributing to mounting of immune responses against anti-onconeural antigens. Taken together, these observations point to increased tumour lymphangiogenesis as a potential contributor to immune tolerance breakdown in PNS. Future studies integrating analysis of primary tumours with their regional lymph nodes, at both the tissue and molecular levels, may provide insight into the sites of antigen presentation and autoimmune priming.

Beyond these shared features, the data presented herein also revealed specific tumour microenvironment-associated gene expression profiles in Yo-BCs compared with Ri-BCs. Yo-BCs displayed a mesenchymal transcriptional profile characterised by the upregulation of EMT-related genes, together with downregulation of *HIF1α* and its target genes. This combination is relatively uncommon, as EMT activation is often associated with hypoxic signalling in most tumour contexts [35, 36]. In Yo-BCs, this dissociation may suggest that mesenchymal reprogramming occurs through alternative pathways. In BCs, non-hypoxic EMT programs have been reported to arise from *HER2*-*PI3K*-*AKT* signalling [37, 38], or from inflammatory pathways activating *NF-κB* and *STAT3* [39, 40]. Consistent with this, the subcategories “*PI3K-AkT* Signalling”, “*NF-κB* Signalling” *and* “*JAK-STAT* Signalling” had higher representation of DE genes in Yo-BCs compared to Ri-BCs. These observations suggest a distinct mode of tumour-stroma crosstalk which may be related to their intrinsic HER2 + background and their intense inflammatory milieu. Whether these features contribute to immune tolerance breakdown remains to be determined.

The present study has several limitations. First, the small cohort size, due to the rarity of PNS, limits the statistical power of the analysis and, consequently, the strength of the conclusions; accordingly, and in light of the results after multiple-testing correction, the present findings should be interpreted as exploratory and hypothesis-generating, and will require validation in larger, independent cohorts. Second, although control tumours were selected based on predefined clinicopathological criteria, residual confounding related to interindividual variability cannot be excluded. In addition, the interpretation of biological pathways and functional processes relies on the predefined annotation framework of the Tumor 360 Signaling Panel (NanoString Technologies), which may have limited the identification of relevant biological axes not captured by this panel.

While partial validation of transcriptomic findings was achieved at the protein level by immunohistochemistry, the limited size of the samples, which led to exhaustion of the available tissue blocks, precluded further analyses of additional markers. In this context, PDPN was selected as a representative marker based on its established reliability in formalin-fixed paraffin-embedded tissues and its well-characterised staining patterns in routine pathology practice, whereas other upregulated candidates (e.g., FLT4, PGF, SPHK2) could not be assessed.

Beyond this limited internal validation, further work is required to establish robust molecular signatures or clinically relevant biomarker candidates associated with PNS-related breast cancer subtypes. Due to the rarity of PNS-associated breast cancers, publicly available datasets are extremely limited, and to our knowledge, no datasets of comparable size are currently available, which restricts opportunities for external validation.

## Conclusion

Increased tumour-associated lymphangiogenesis was identified as a common feature in BCs linked to PNSs. Vascular remodelling, together with transcriptional features associated with invasiveness, may facilitate lymph-node dissemination of tumour cells carrying neoantigens, ultimately promoting their presentation and contributing to the immune tolerance breakdown observed in PNS. These observations raise the possibility that the route and context of antigen exposure may be critical in PNS pathophysiology and support further investigation of lymphatic dissemination and lymph node compartments.

## Supplementary Information

Below is the link to the electronic supplementary material.

Supplementary Material 1.

Supplementary Material 2.

Supplementary Material 3.

Supplementary Material 4.

Supplementary Material 5.

Supplementary Material 6.

Supplementary Material 7.

Supplementary Material 8.

Supplementary Material 9.

Supplementary Material 10.

Supplementary Material 11.

## Data Availability

The datasets used and/or analysed during the current study are available from the corresponding author on reasonable request.

## Abbreviations

PNS: Paraneoplastic neurological syndrome
BC: Breast cancer
Yo-BC: Breast cancer associated with Yo paraneoplastic neurological syndrome
Ri-BC: Breast cancer associated with Ri paraneoplastic neurological syndrome
CtrlYo: Control breast cancer matched with breast cancer associated with Yo
CtrlRi: Control breast cancer matched with breast cancer associated with Ri
HER2 +: Human epidermal growth factor receptor 2 positive
HR-: Hormone receptor negative
CA IX: Carbonic anhydrase IX
PDPN: Podoplanin
DE: Differentially expressed
