# Supplementary material for: Vascular and mesenchymal signatures of breast cancers associated with Yo and Ri paraneoplastic syndrome
Source: Breast Cancer Res. 2026 Jun 17;28:148. doi: 10.1186/s13058-026-02321-9 (PMC13508272; doi:10.1186/s13058-026-02321-9)
Supplement: Supplementary file 1 [file 13058_2026_2321_MOESM10_ESM.docx]

**Figure S3. Functional annotation of differentially expressed genes across immune and tumour signalling domains in Yo-BC, Ri-BC compared to respective controls**. (a) Histograms showing the number of DE genes annotated to the immune (upper panel) and tumour signalling (lower panel) domains in Yo-BC vs CtrlYo and Ri-BC vs CtrlRi comparisons. Genes were grouped in categories (in bold) and subcategories (in italics). Asterisks indicate categories or subcategories showing significant differences in the number of DE genes between the two comparisons (*****p < 0·05; ******p < 0·01; *******p < 0·001; ********p < 0·0001; Fisher’s exact test). (b) Immune cell-type signatures differentially expressed in Yo-BC and Ri-BC compared to their controls. DE = differentially expressed.
