# Supplementary material for: Vascular and mesenchymal signatures of breast cancers associated with Yo and Ri paraneoplastic syndrome
Source: Breast Cancer Res. 2026 Jun 17;28:148. doi: 10.1186/s13058-026-02321-9 (PMC13508272; doi:10.1186/s13058-026-02321-9)
Supplement: Supplementary file 2 [file 13058_2026_2321_MOESM11_ESM.docx]

**Figure S4. Hypoxia-related signals in Ri-BC and CtrlRi.** (a) Normalised mRNa expression counts for genes belonging to the HIF1 Signalling subcategory (NOS3 and RBX1) in Ri-BC vs CtrlRi comparison. Box-and-whisker plots show median, minimum to maximum, and individual sample values (*p < 0·05; Wilcoxon-Mann-Whitney test). (b) Histograms showing CA IX staining quantification in Ri-BC compared to CtrlRi. CA IX was quantified by immunohistochemistry using a semi-quantitative additive score, combining staining intensity on tumour cells and myofibroblast staining extent. Tumours were classified as CA IX-high (CA IX score ≥2) or CA IX-low. (c) Representative images showing immunohistochemical detection of CA IX in Ri-BC (n=10) and CtrlRi (n=7). Insets illustrate high-magnification views where CA IX expression is observed in tumour cells of Ri and not in CtrlRi. Scale bars: main images, 100 µm; insets, 50 µm. BC, breast cancer; CA IX, carbonic anhydrase 9; NOS3, nitric oxide synthase 3; RBX1, ring-box 1.
