# Supplementary material for: Vascular and mesenchymal signatures of breast cancers associated with Yo and Ri paraneoplastic syndrome
Source: Breast Cancer Res. 2026 Jun 17;28:148. doi: 10.1186/s13058-026-02321-9 (PMC13508272; doi:10.1186/s13058-026-02321-9)
Supplement: Supplementary file 3 [file 13058_2026_2321_MOESM1_ESM.docx]

|  | | **Yo-BCs (n=12), n (%)** | **CtrlYo BCs (n=18), n (%)** |
| --- | --- | --- | --- |
| **Tumour subtype** | | | |
|  | HER2- HR+ | 2 (16·6) | 2 (11·1) |
|  | HER2+ HR+ | 1 (8·3) | 0 |
|  | HER2+ HR- | 6 (50) | 13 (72·2) |
|  | TNBC | 2 (16·6) | 1 (5·5) |
|  | NA | 1 (8·3) | 2 (11·1) |
| **Lymph node metastasis** |  |  |  |
|  | N0 | 5 (41·6) | 5 (27·7) |
|  | N+ | 7 (58·3) | 13 (72·2) |
|  | | | |
|  |  | **Ri-BCs (n=10), n (%)** | **CtrlRi BCs (n=8), n (%)** |
| **Tumour subtype** | | | |
|  | HER2- HR+ | 7 (70) | 5 (62·5) |
|  | HER2+ HR+ | 3 (30) | 3 (37·5) |
|  | HER2+ HR- | 0 (0) | 0 (0) |
|  | TNBC | 0 (0) | 0 (0) |
|  | NA | 0 (0) | 0 (0) |
| **Lymph node metastasis** |  |  |  |
|  | N0 | 4 (40) | 3 (37·5) |
|  | N+ | 6 (60) | 5 (62·5) |

**Table S1. Clinicopathologic description of breast cancer samples in the Yo-PNS and Ri-PNS cohorts. and controls.** Data are n (%). Tumour subtype classification is based on HER2 and HR status. Lymph node metastasis status: N0, no metastasis; N+ positive lymph nodes. Percentages are calculated relative to the total number of BC cases in each group. BC, breast cancer; HER2, human epidermal growth factor receptor 2; HR, hormone receptor; NA, not available; TNBC, triple-negative breast cancer.
