# Supplementary material for: Vascular and mesenchymal signatures of breast cancers associated with Yo and Ri paraneoplastic syndrome
Source: Breast Cancer Res. 2026 Jun 17;28:148. doi: 10.1186/s13058-026-02321-9 (PMC13508272; doi:10.1186/s13058-026-02321-9)
Supplement: Supplementary file 4 [file 13058_2026_2321_MOESM2_ESM.docx]

| **Nanostring Samples** |  | **Yo-BCs (n=10), n (%)** | **CtrlYo BCs (n=10), n (%)** |
| --- | --- | --- | --- |
| **Tumour Subtype** | HER2-HR+ | 2 (20·0) | 1 (10·0) |
|  | HER2+HR+ | 1 (10·0) | 0 (0) |
|  | HER2+HR- | 4 (40·0) | 7 (70·0) |
|  | TNBC | 2 (20·0) | 0 (0) |
|  | NA | 1 (10·0) | 2 (20·0) |
| **Lymph node metastasis** |  |  |  |
|  | N0 | 5 (50·0) | 3 (30·0) |
|  | N+ | 5 (50·0) | 7 (70·0) |
|  |  |  |  |
|  |  | **Ri-BCs (n=8), n (%)** | **CtrlRi BCs (n=5), n (%)** |
| **Tumour Subtype** | HER2-HR+ | 6 (75·0) | 3 (60·0) |
|  | HER2+HR+ | 2 (25·0) | 2 (40·0) |
|  | HER2+HR- | 0 (0) | 0 (0) |
|  | TNBC | 0 (0) | 0 (0) |
|  | NA | 0 (0) | 0 (0) |
| **Lymph node metastasis** |  |  |  |
|  | N0 | 3 (37·5) | 3 (60·0) |
|  | N+ | 5 (62·5) | 2 (40·0) |

| **IHC Samples** |  | **Yo-BCs (n=8), n (%)** | **CtrlYo BCs (n=9), n (%)** |
| --- | --- | --- | --- |
| **Tumour Subtype** | HER2-HR+ | 0 (0) | 0 (0) |
|  | HER2+HR+ | 1 (12·5) | 0 (0) |
|  | HER2+HR- | 4 (50·0) | 9 (100·0) |
|  | TNBC | 2 (25·0) | 0 (0) |
|  | NA | 1 (12·5) | 0 (0) |
| **Lymph node metastasis** |  |  |  |
|  | N0 | 3 (37·5) | 4 (44·4) |
|  | N+ | 5 (62·5) | 5 (55·5) |
|  |  |  |  |
|  |  | **Ri-BCs (n=10), n (%)** | **CtrlRi BCs (n=7), n (%)** |
| **Tumour Subtype** | HER2-HR+ | 7 (70·0) | 4 (57·1) |
|  | HER2+HR+ | 3 (30·0) | 3 (42·8) |
|  | HER2+HR- | 0 (0) | 0 (0) |
|  | TNBC | 0 (0) | 0 (0) |
|  | NA | 0 (0) | 0 (0) |
| **Lymph node metastasis** |  |  |  |
|  | N0 | 3 (30·0) | 3 (42·8) |
|  | N+ | 4 (40·0) | 4 (57·1) |

**Table S2. Clinicopathologic description of breast cancer samples in the NanoString subset and the IHC subset.** Data are n (%). Tumour subtype classification is based on HER2 and HR status. Lymph node metastasis status: N0, no metastasis; N+ positive lymph nodes. Percentages are calculated relative to the total number of BC cases in each group. BC, breast cancer; HER2, human epidermal growth factor receptor 2; HR, hormone receptor; NA, not available; TNBC, triple-negative breast cancer.
