# Supplementary material for: Vascular and mesenchymal signatures of breast cancers associated with Yo and Ri paraneoplastic syndrome
Source: Breast Cancer Res. 2026 Jun 17;28:148. doi: 10.1186/s13058-026-02321-9 (PMC13508272; doi:10.1186/s13058-026-02321-9)
Supplement: Supplementary file 5 [file 13058_2026_2321_MOESM4_ESM.docx]

| **Genes** | **Annotation** |
| --- | --- |
| *ADORA2A* | Immune Response |
| *ARG1* | Immune Response |
| *COP1* | Tumour Signalling |
| *COX5B* | Tumour Signalling |
| *CRK* | Tumour Signalling |
| *CXCR2* | Immune Response |
| *EIF4G1* | Tumour Signalling |
| *FLT4* | Microenvironment |
| *GLUD2* | Tumour Signalling |
| *HRAS* | Tumour Signalling |
| *IQGAP1* | Tumour Signalling |
| *KLK2* | Microenvironment |
| *MAFG* | Tumour Signalling |
| *PA2G4* | Tumour Signalling |
| *PDCD1* | Immune Response |
| *PGF* | Microenvironment |
| *PIK3CB* | Tumour Signalling |
| *PNOC* | Immune Response |
| *POU5F1* | Tumour Signalling |
| *RPTOR* | Tumour Signalling |
| *SIN3A* | Tumour Signalling |
| *SLC2A1* | Tumour Signalling |
| *SNAI1* | Microenvironment |
| *SPHK2* | Microenvironment |
| *SQSTM1* | Tumour Signalling |
| *SRC* | Tumour Signalling |
| *UCHL5* | Tumour Signalling |

**Table S5**. **Manual re-assignment of the 27 shared DE genes**.
