# Supplementary material for: Vascular and mesenchymal signatures of breast cancers associated with Yo and Ri paraneoplastic syndrome
Source: Breast Cancer Res. 2026 Jun 17;28:148. doi: 10.1186/s13058-026-02321-9 (PMC13508272; doi:10.1186/s13058-026-02321-9)
Supplement: Supplementary file 6 [file 13058_2026_2321_MOESM6_ESM.docx]

| **Level of annotation** | **Annotation** | **Number of genes, n (%)** | **Genes** |
| --- | --- | --- | --- |
| **Domain** | **Tumour Signalling** |  |  |
| Category | Cellular Energetics | 26 (19·8%) | ***MAFG***, ***RPTOR***, ***UCHL5***, ***SQSTM1***, ***GLUD2***, ***COX5B***, ***SLC2A1***, *LDHA*, *NDUFB1*, *STAT3*, *PRDX2*, *KEAP1*, *GABPA*, *NCF1*, *RB1*, *PIK3R4*, *AKT1S1*, *RICTOR*, *CHMP2A*, *ERN1*, *P4HB*, *ERO1A*, *XBP1*, *RB1CC1*, *ELOVL5*, *SQLE* |
| Subcategory | Autophagy | 6 (4·6%) | ***RPTOR***, ***SQSTM1***, *PIK3R4*, *CHMP2A*, *ERN1*, *RB1CC1* |
| Subcategory | Glucose Metabolism | 6 (4·6%) | ***GLUD2***, ***COX5B***, ***SLC2A1***, *LDHA*, *NDUFB1*, *GABPA* |
| Subcategory | Glutamine Metabolism | 1 (7·6%) | ***GLUD2*** |
| Subcategory | Lipid Metabolism | 0 |  |
| Subcategory | mTOR Signalling | 12 (9·2%) | ***RPTOR***, ***UCHL5***, ***EIF4G***, ***SQSTM1***, ***SLC2A1***, *LDHA*, *AKT1S1*, *RICTOR*, *ERO1A*, *XBP1*, *ELOVL5*, *SQLE* |
| Subcategory | Nrf2 & Oxidative Stress | 7 (5·3%) | ***MAFG***, *PRDX2*, *KEAP1*, *GABPA*, *NCF1*, *P4HB*, *ERO1A* |
| Category | Sustained Proliferation | 45 (34·4%) | ***SRC***, ***KLK2***, ***CRK***, ***PA2G4***, ***PIK3CB***, ***IQGAP1***, ***HRAS***, *VWF*, *ACTG2*, *TBRG4*, *ABL1*, *CALML3*, *PTCH2*, *FZD4*, *EGF*, *ESRP2*, *CDCP1*, *PDGFRB*, *TGFB1*, *TGFB3*, *FGFR3*, *HIF1A*, *LDHA*, *STAT3*, *JAK3*, *JAK2*, *CSF2RB*, *CCNT1*, *CDK1*, *ESR1*, *TFDP1*, *GNAI3*, *NOTCH3*, *PIK3R2*, *PIK3R4*, *PIK3CD*, *AKT1S1*, *KAT2B*, *KDM1A*, *PPAN*, *NKX3-1*, *P4HB*, *ELOVL5*, *ATF4*, *SMC3* |
| Subcategory | Androgen Signalling | 4 (3·1%) | ***KLK2***, *RB1*, *NKX3-1*, *ELOVL5* |
| Subcategory | EGFR Signalling | 3 (2·3%) | **HRAS**, **SRC**, EGF |
| Subcategory | ERBB2 Signalling | 3 (2·3%) | **HRAS**, **SRC**, EGF |
| Subcategory | Oestrogen Signalling | 8 (6·1%) | *CALML3*, *CCNT1*, *ESR1*, *GNAI3*, *KAT2B*, *KDM1A*, *ATF4*, *SMC3* |
| Subcategory | FGFR Signalling | 3 (2·3%) | ESRP2, FGFR3, **HRAS** |
| Subcategory | Hedgehog | 3 (2·3%) | *P4HB*, *ABL1*, *PTCH2* |
| Subcategory | MAPK Signalling | 11 (8·4%) | ***SRC***, ***IQGAP1***, ***HRAS***, *VWF*, *EGF*, *CDCP1*, *PDGFRB*, *JAK3*, *JAK2*, *CSF2RB*, *CDK1* |
| Subcategory | MET Signalling | 4 (3·1%) | ***SRC***, ***CRK***, ***HRAS***, *STAT3* |
| Subcategory | Myc | 4 (3·1%) | ***PA2G4***, *TBRG4*, *LDHA*, *PPAN* |
| Subcategory | Notch Signalling | 2 (1·5%) | *NOTCH3*, *KAT2B* |
| Subcategory | PI3K-Akt Signalling | 6 (4·5%) | ***PIK3CB***, *PIK3R2*, *PIK3R4*, *PIK3CD*, *AKT1S1*, *ATF4* |
| Subcategory | TGF-beta Signalling | 5 (3·8%) | ***IQGAP1***, *ACTG2*, *TGFB1*, *TGFB3*, *TFDP1* |
| Subcategory | Wnt Signalling | 1 (0·7%) | *FZD4* |
| Category | Evading Growth Suppressors | 14 (10·6%) | ***COP1***, *CCNT1*, *CDK1*, *CDKN2A*, *CCNB2*, *NUF2*, *TFDP1*, *TTK*, *SKA1*, *RB1*, *RFC3*, *DKC1*, *SMC3*, *PRIM1* |
| Subcategory | Cell Cycle | 14 (10·6%) | ***COP1***, *CCNT1*, *CDK1*, *CDKN2A*, *CCNB2*, *NUF2*, *TFDP1*, *TTK*, *SKA1*, *RB1*, *RFC3*, *DKC1*, *SMC3*, *PRIM1* |
| Category | Enabling Replicative Immortality | 8 (6·1%) | ***POU5F1***, *STAT3*, *CDKN2A*, *POLE*, *RB1*, *RFC3*, *DKC1*, *PRIM1* |
| Subcategory | Immortality & Stemness | 7 (5·3%) | ***POU5F1***, *STAT3*, *POLE*, *RB1*, *DKC1*, *RFC3*, *PRIM1* |
| Subcategory | Senescence | 1 (0·8%) | *CDKN2A* |
| Category | Genomic Instability | 17 (12·9%) | ***SIN3A***, ***COP1***, *TRAF1*, *CCNT1*, *CHEK1*, *POLE*, *KAT6B*, *NSD1*, *SUPT16H*, *KAT2B*, *KMT2D*, *KDM1A*, *KDM5A*, *KDM6A*, *ATF4*, *MLH1*, *MSH2* |
| Subcategory | DNA Damage Repair | 4 (3·1%) | *CHEK1*, *POLE*, *MLH1*, *MSH2* |
| Subcategory | Epigenetic & Transcriptional Regulation | 11 (8·4%) | ***SIN3A***, *TRAF1*, *CCNT1*, *KAT6B*, *NSD1*, *KAT2B*, *KMT2D*, *KDM1A*, *KDM5A*, *KDM6A*, *ATF4* |
| Subcategory | p53 Signalling | 3 (2·3%) | ***COP1***, *CHEK1*, *SUPT16H* |
| Category | Resisting Cell Death | 4 (3·1%) | *CD27*, *TNF*, *TNFSF14*, *BAD* |
| Subcategory | Apoptosis | 1 (0·8%) | BAD |
| Subcategory | TNF Superfamily | 3 (2·3%) | TNF, TNFSF14, CD27 |
| Domain | Immune Response |  |  |
| Category | Immune Evasion | 17 (12·9%) | ***ARG1***, ***ADORA2***, ***PDCD1***, *HIF1A*, *CD209*, *CD27*, *CD40LG*, *CTSW*, *FCRL2*, *KLRB1*, *PTPN6*, *LAT*, *TOX*, *ZAP70*, *TNF*, *RB1*, *GRAP2* |
| Subcategory | Antigen Presentation | 0 |  |
| Subcategory | B-Cell Function | 1 (0·8%) | *FCRL2* |
| Subcategory | Cytotoxicity | 2 (1·5%) | *CTSW*, *KLRB1* |
| Subcategory | Myeloid Immune Evasion | 3 (2·3%) | ***ARG1***, ***ADORA2***, *CD209* |
| Subcategory | T-cell Costimulation | 4 (3·1%) | ***ADORA2***, ***PDCD1***, *CD27*, *CD40LG* |
| Subcategory | T-cell Exhaustion | 3 (2·3%) | *HIF1A*, ***PDCD1***, *TOX* |
| Subcategory | TCR Signalling | 6 (4·6%) | ***PDCD1***, *CD40LG*, *PTPN6*, *LAT*, *TNF*, *GRAP2* |
| Subcategory | Tumour Antigen | 0 |  |
| Category | Tumour Promoting Inflammation | 30 (22·9%) | ***CXCR2***, ***CRK***, ***PA2G4***, ***EIF4G1***, ***SQSTM1***, *DOCK2*, *STAT3*, *PTGER4*, *CD40LG*, *JAK3*, *PTPN6*, *LAT*, *IRF4*, *IFNAR2*, *JAK2*, *CHUK*, *PLCG2*, *FCGRA/B*, *ZAP70*, *LTB*, *CCL21*, *CCR2*, *CSF2RB*, *TNF*, *TNFSF14*, *TRAF1*, *TRAF6*, *MCL1*, *CFLAR*, *GNAI3* |
| Subcategory | Chemokine Signalling | 3 (2·3%) | *CCL21*, ***CXCR2***, *CCR2* |
| Subcategory | Inflammation | 6 (4·5%) | ***PA2G4***, *DOCK2*, *PTGER4*, *FCGRA/B*, *LTB*, *GNAI3* |
| Subcategory | Interferon Response | 4 (3·1%) | ***EIF4G1***, *PTPN6*, *IRF4*, *IFNAR2* |
| Subcategory | Interleukin Signalling | 3 (2·3%) | ***CRK***, *CSF2RB*, *CSF2RB* |
| Subcategory | JAK-STAT Signalling | 5 (3·8%) | *STAT3*, *JAK3*, *JAK2*, *PTPN6*, *MCL1* |
| Subcategory | NF-kB Signalling | 13 (9·9%) | ***SQSTM1***, *CD40LG*, *LAT*, *CHUK*, *PLCG2*, *ZAP70*, *LTB*, *CCL21*, *TNF*, *TNFSF14*, *TRAF1*, *TRAF6*, *CFLAR* |
| Category | Immune cells | 8 (6·1%) | *FCRA/B*, *CD209*, *CTSW*, *KLRB1*, *FCRL2*, ***PNOC***, *FAM30A*, *PTGER4* |
| Subcategory | Tregs | 0 |  |
| Subcategory | Neutrophils | 1 (0·8%) | *FCGRA/B* |
| Subcategory | DCs | 1 (0·8%) | *CD209* |
| Subcategory | Cytotoxic T Cells | 2 (1·5%) | *CTSW*, *KLRB1* |
| Subcategory | B Cells | 3 (2·3%) | *FCRL2*, ***PNOC***, *FAM30A* |
| Subcategory | NK CD56dim Cells | 0 |  |
| Subcategory | Mast Cells | 0 |  |
| Subcategory | Exhausted CD8 | 1 (0·8%) | *PTGER4* |
| Domain | Microenvironment |  |  |
| Category | Activating Invasion and Metastasis | 20 (15·2%) | ***FLT4***, ***SRC***, ***PGF***, ***SNAI1***, ***KLK2***, ***POU5F1***, *VWF*, *EGF*, *CTNNA1*, *TGFB1*, *TGFB3*, *HIF1A*, *STAT3*, *JAK2*, *CCL21*, *TNF*, *ESR1*, *LATS1*, *PDPK1*, *KDM1A* |
| Subcategory | Cell Adhesion & Motility | 4 (3·1%) | *PDPK1*, ***FLT4***, ***PGF***, *CTNNA1* |
| Subcategory | ECM Remodelling & Metastasis | 4 (3·1%) | *VWF*, ***KLK2***, *TGFB1*, *TGFB3* |
| Subcategory | EMT | 11 (8·4%) | ***SRC***, ***SNAI1***, ***POU5F1***, *EGF*, *TGFB1*, *HIF1A*, *STAT3*, *JAK2*, *CCL21*, *ESR1*, *KDM1A*, |
| Subcategory | Hippo Signalling | 2 (1·5%) | *CTNNA1*, *LATS1* |
| Category | Angiogenesis | 16 (12·2%) | ***FLT4***, ***SRC***, ***CRK***, ***PIK3CB***, ***SLC2A1***, ***SPHK2***, ***HRAS***, *FLT1*, *VEGFD*, *P4HA2*, *PDGFRB*, *HIF1A*, *LDHA*, *STAT3*, *PLCG2*, *PIK3R2* |
| Subcategory | HIF1 Signalling | 6 (4·6%) | ***SLC2A1***, *FLT1*, *P4HA2*, *TGFB3*, *HIF1A*, *LDHA* |
| Subcategory | PDGF Signalling | 7 (5·3%) | ***SRC***, ***CRK***, ***PIK3CB***, ***HRAS***, *PDGFRB*, *STAT3*, *PIK3R2* |
| Subcategory | VEGF Signalling | 5 (3·8%) | ***FLT4***, ***SPHK2***, *FLT1*, *VEGFD*, *PLCG2*, |

**Table S7**. **Distribution of differentially expressed genes in Yo-BC compared to CtrlYo according to the functional classification provided by NanoString**. The table presents DE genes assigned to each annotation level: Domain, Category, and Subcategory. Number of genes, n (%) reports the number and proportion of DE gene’s annotations within each group, calculated relative to the total number of DE genes in Yo-BC vs CtrlYo (n = 131). The column ‘Genes’ lists the individual DE genes assigned to each annotation group. Bold indicates DE genes shared genes between the two comparisons (Yo-BC vs CtrlYo and Ri-BC vs CtrlRi). BC, breast cancer; DE, differentially expressed.
