# Supplementary material for: Vascular and mesenchymal signatures of breast cancers associated with Yo and Ri paraneoplastic syndrome
Source: Breast Cancer Res. 2026 Jun 17;28:148. doi: 10.1186/s13058-026-02321-9 (PMC13508272; doi:10.1186/s13058-026-02321-9)
Supplement: Supplementary file 7 [file 13058_2026_2321_MOESM7_ESM.docx]

| **Annotation level** | **Annotation** | **Number of genes, n (%)** | **Genes** |
| --- | --- | --- | --- |
| **Domain** | **Tumour signalling** |  |  |
| Category | Cellular Energetics | 13 (17·3%) | ***MAFG***, ***RPTOR***, ***UCHL5***, ***SQSTM1***, ***GLUD2***, ***COX5B***, ***SLC2A1***, *CDC25A*, *MCM2*, *MTOR*, *LAMTOR4*, *ATG4B*, *ACSL3* |
| Subcategory | Autophagy | 4 (5·3%) | ***RPTOR***, ***SQSTM1***, *MTOR*, *ATG4B* |
| Subcategory | Glucose Metabolism | 3 (4%) | ***GLUD2***, ***COX5B***, ***SLC2A1*** |
| Subcategory | Glutamine Metabolism | 1 (1·3%) | ***GLUD2*** |
| Subcategory | Lipid Metabolism | 0 |  |
| Subcategory | mTOR Signalling | 9 (12%) | ***RPTOR***, ***UCHL5***, ***EIF4G***, ***SQSTM1***, ***SLC2A1***, *CDC25A*, *MCM2*, *MTOR*, *LAMTOR4*, *ACSL3* |
| Subcategory | Nrf2 & Oxidative Stress | 1 (1·3%) | ***MAFG*** |
| Category | Sustained Proliferation | 21 (28%) | ***SRC***, ***KLK2***, ***CRK***, ***PA2G4***, ***PIK3CB***, ***IQGAP1***, ***HRAS***, *NOS3*, *KLK3*, *TNS4*, *BMPR2*, *CCNE1*, *RBX1*, *RBL1*, *AGO2*, *RBBP5*, *ACSL3*, *SRM*, *TCOF1*, *DUSP6*, *MAP2K1* |
| Subcategory | Androgen Signalling | 4 (5·3%) | ***KLK2***, *KLK3*, *CCNE1*, *ACSL3* |
| Subcategory | EGFR Signalling | 2 (2·6%) | ***HRAS***, ***SRC*** |
| Subcategory | ERBB2 Signalling | 2 (2·6%) | ***HRAS***, ***SRC*** |
| Subcategory | Oestrogen Signalling | 2 (2·6%) | *NOS3*, *AGO2* |
| Subcategory | FGFR Signalling | 1 (1·3%) | ***HRAS*** |
| Subcategory | Hedgehog | 0 |  |
| Subcategory | MAPK Signalling | 8 (10·6%) | ***SRC***, ***IQGAP1***, ***HRAS***, *RBX1*, *AGO2*, *DUSP6*, *MAP2K1*, *KRAS* |
| Subcategory | MET Signalling | 4 (5·3%) | ***SRC***, ***CRK***, ***HRAS***, *TNS4* |
| Subcategory | Myc | 4 (5·3%) | ***PA2G4***, *CCNE1*, *SRM*, *TCOF1* |
| Subcategory | Notch Signalling | 0 |  |
| Subcategory | PI3K-Akt Signaling | 1 (1·3%) | ***PIK3CB*** |
| Subcategory | TGF-beta Signalling | 3 (4%) | ***IQGAP1***, *BMPR2*, *RBL1* |
| Subcategory | Wnt Signalling | 1 (1·3%) | *RBBP5* |
| Category | Evading Growth Suppressors | 11 (14·6%) | ***COP1***, *CDC25A*, *CCNE1*, *E2F2*, *MCM2*, *RBX1*, *RBL1*, *MYBL2*, *CCNK*, *LIG1*, *RPA3* |
| Subcategory | Cell cycle | 11 (14·6%) | ***COP1***, *CDC25A*, *CCNE1*, *E2F2*, *MCM2*, *RBX1*, *RBL1*, *MYBL2*, *CCNK*, *LIG1*, *RPA3* |
| Category | Enabling Replicative Immortality | 5 (6·6%) | ***POU5F1***, *CCNE1*, *LIG1*, *RPA3*, *KRAS* |
| Subcategory | Immortality & Stemness | 3 (4%) | ***POU5F1***, *LIG1*, *RPA3* |
| Subcategory | Senescence | 2 (2·6%) | *CCNE1*, *E2F2* |
| Category | Genomic Instability | 9 (12%) | ***SIN3A***, ***COP1***, *ARID4B*, *AGO2*, *CCNK*, *RBBP5*, *BIRC3*, *LIG1*, *H2AX* |
| Subcategory | DNA Damage Repair | 3 (4%) | *LIG1*, *H2AX*, *RPA3* |
| Subcategory | Epigenetic & Transcriptional Regulation | 5 (6·6%) | ***SIN3A***, *ARID4B*, *AGO2*, *RBBP5*, *BIRC3* |
| Subcategory | p53 Signalling | 2 (2·6%) | ***COP1***, *CCNK* |
| Category | Resisting cell death | 3 (4%) | *GZMB*, *TNFRSF18*, *TNFRSF4* |
| Subcategory | Apoptosis | 1 (1·3%) | *GZMB* |
| Subcategory | TNF Superfamily | 2 (2·6%) | *TNFRSF18*, *TNFRSF4* |
| Domain | Immune Response |  |  |
| Category | Immune Evasion | 16 (21·3%) | ***ARG1***, ***ADORA2***, ***PDCD1***, *KLK3*, *CEACAM1*, *IDO1*, *KLRD1*, *TAP1*, *MAGEC1*, *GZMB*, *TOX2*, *ICOS*, *KIR3DL2*, *IFNG*, *TNFRSF18*, *TNFRSF4* |
| Subcategory | Antigen Presentation | 1 (1·3%) | *TAP1* |
| Subcategory | B-cell Function | 0 |  |
| Subcategory | Cytotoxicity | 3 (4%) | *KLRD1*, *GZMB*, *KIR3DL2* |
| Subcategory | Myeloid Immune Evasion | 3 (4%) | ***ARG1***, ***ADORA2***, *IDO1* |
| Subcategory | T-cell Costimulation | 6 (8%) | ***ADORA2***, ***PDCD1***, *FOXP3*, *ICOS*, *TNFRSF18*, *TNFRSF4* |
| Subcategory | T-cell Exhaustion | 2 (2·6%) | ***PDCD1***, *TOX2* |
| Subcategory | TCR Signalling | 3 (4%) | ***PDCD1***, *ICOS*, *IFNG* |
| Subcategory | Tumour Antigen | 3 (4%) | *KLK3*, *CEACAM1*, *MAGEC1* |
| Category | Tumour Promoting Inflammation | 13 (17·3%) | ***CXCR2***, ***CRK***, ***PA2G4***, ***EIF4G1***, ***SQSTM1***, *CEACAM1*, *IRF1*, *NFKB2*, *IL15RA*, *IFNG*, *IL12B*, *IL7*, *BIRC3* |
| Subcategory | Chemokine Signalling | 1 (1·3%) | ***CXCR2*** |
| Subcategory | Inflammation | 2 (2·6%) | ***PA2G4***, *CEACAM1* |
| Subcategory | Interferon Response | 3 (4%) | ***EIF4G1***, *IRF1*, *IFNG* |
| Subcategory | Interleukin Signalling | 4 (5·3%) | ***CRK***, *IL15RA*, *IL12B*, *IL7* |
| Subcategory | JAK-STAT Signalling | 0 |  |
| Subcategory | NF-kB Signalling | 3 (4%) | ***SQSTM1***, *NFKB2*, *BIRC3* |
| Category | Immune cells | 8 (6·6 %) | *FOXP3*, *KLRD1*, *KI3DL2*, *HDC*, ***PNOC*** |
| Subcategory | Tregs | 1 (1·3%) | *FOXP3* |
| Subcategory | Neutrophils | 0 |  |
| Subcategory | DCs | 0 |  |
| Subcategory | Cytotoxic T Cells | 1 (1·3%) | *KLRD1* |
| Subcategory | B Cells | 1 (1·3%) | ***PNOC*** |
| Subcategory | NK CD56dim Cells | 1 (1·3%) | *KIR3DL2* |
| Subcategory | Mast Cells | 1 (1·3%) | *HDC* |
| Subcategory | Exhausted CD8 | 0 |  |
| Domain | Microenvironment |  |  |
| Category | Activating Invasion and Metastasis | 10 (13·2%) | ***FLT4***, ***SRC***, ***PGF***, ***SNAI1***, ***KLK2***, ***POU5F1***, *CEACAM1*, *MMP9*, *MTOR*, *KRAS* |
| Subcategory | Cell Adhesion & Motility | 2 (2·6%) | ***FLT4***, ***PGF*** |
| Subcategory | ECM Remodelling & Metastasis | 3 (4%) | ***KLK2***, *CEACAM1*, *MMP9* |
| Subcategory | EMT | 4 (5·3%) | ***SRC***, ***SNAI1***, ***POU5F1***, *KRAS* |
| Subcategory | Hippo Signalling | 1 (1·3%) | *KRAS* |
| Category | Angiogenesis | 9 (12%) | ***FLT4***, ***SRC***, ***CRK***, ***PIK3CB***, ***SLC2A1***, ***SPHK2***, ***HRAS***, *NOS3*, *RBX1* |
| Subcategory | HIF1 Signalling | 3 (4%) | ***SLC2A1***, *NOS3*, *RBX1* |
| Subcategory | PDGF Signalling | 4 (5·3%) | ***SRC***, ***CRK***, ***PIK3CB***, ***HRAS*** |
| Subcategory | VEGF Signalling | 5 (3·8%) | ***FLT4***, ***SPHK2***, *NOS3* |

**Table S8**. **Distribution of differentially expressed genes in Ri-BC compared to CtrlRi according to the functional classification provided by NanoString**. Annotation level indicates the hierarchy **(**Domain, Category, and/or Subcategory). Number of genes, n (%) reports the number and proportion of DE gene’s annotations within each group, calculated relative to the total number of DE genes in Ri-BC vs CtrlRi (n=75). The column ‘Genes’ lists the individual DE genes assigned to each annotation group. Bold indicates DE genes shared genes between the two comparisons (Yo-BC vs CtrlYo and Ri-BC vs CtrlRi). BC, breast cancer; DE, differentially expressed.
