# Supplementary material for: Vascular and mesenchymal signatures of breast cancers associated with Yo and Ri paraneoplastic syndrome
Source: Breast Cancer Res. 2026 Jun 17;28:148. doi: 10.1186/s13058-026-02321-9 (PMC13508272; doi:10.1186/s13058-026-02321-9)
Supplement: Supplementary file 8 [file 13058_2026_2321_MOESM8_ESM.docx]

**Figure S1.** **Expression of shared differentially expressed genes.** Heatmap presenting the z-score transformed expression levels of the 27 shared DE genes in Yo-BC vs CtrlYo and Ri-BC vs CtrlRi. Genes are organised in ascending order based on the absolute value of their log2 fold change assessed in the comparison Yo-BC vs CtrlYo. DE, differentially expressed.
