# Supplementary material for: Vascular and mesenchymal signatures of breast cancers associated with Yo and Ri paraneoplastic syndrome
Source: Breast Cancer Res. 2026 Jun 17;28:148. doi: 10.1186/s13058-026-02321-9 (PMC13508272; doi:10.1186/s13058-026-02321-9)
Supplement: Supplementary file 9 [file 13058_2026_2321_MOESM9_ESM.docx]

**Figure S2. Correlation between PDPN semiquantitative score and FLT4 gene expression.** PDPN expression was assessed using a semi-quantitative score (0-3) based on the number of PDPN+ capillaries. FLT4 transcript levels were measured using NanoString analysis (normalised mRNa expression counts). Each dot represents an individual sample from Yo-BC, Ri-BC, or respective control groups (*****p < 0·05; Spearman’s correlation). FLT4, Fms-related tyrosine kinase 4; PDPN, podoplanin.
